# A poisson flow-based data augmentation and lightweight diagnosis framework for imbalanced rolling bearing faults

**DOI:** 10.1371/journal.pone.0332994

**Published:** 2025-10-06

**Authors:** Xin Liu, Han Wang, Zhiyong Du, Xu Xu, Bo Song

**Affiliations:** 1 CHN Energy BaoRiXiLe Energy Co., Ltd., Hulunbuir, China; 2 CCTEG Shenyang Engineering Company, Shenyang, China; The Hong Kong Polytechnic University, CHINA

## Abstract

Accurate diagnosis of rolling bearing faults is vital for the safe operation of rotating machinery. However, real-world fault datasets often suffer from severe class imbalance, which hinders the performance of deep learning models. To address this challenge, we propose PFRNet, a novel diagnostic framework integrating a Poisson Flow-based generative model with a lightweight residual network. Raw vibration signals are transformed into time-frequency representations via CWT to capture non-stationary fault features. The Poisson generative mechanism models sample evolution in high-dimensional latent space to synthesize realistic minority-class samples by learning statistical distributions of real data, mitigating imbalance. These augmented datasets are subsequently classified using an efficient residual network designed for robust feature extraction with minimal complexity. Experiments on the CWRU benchmark demonstrate that PFRNet outperforms state-of-the-art methods in diagnostic accuracy, robustness, and generalization across various imbalance scenarios. Quantitative evaluations further confirm that the generated samples closely resemble real data in both quality and diversity, supporting the effectiveness of the proposed method. The proposed approach offers a promising solution for reliable fault diagnosis under practical, imbalance-prone industrial conditions.

## 1. Introduction

In the field of high-end equipment manufacturing, condition monitoring and damage identification of core components in rotating machinery have become critical technical challenges for ensuring equipment reliability. As the central hub of power transmission in rotating machinery, the operational performance of rolling bearings directly affects the continuous operation of major equipment such as shield tunneing systems and wind turbines. Bearing components operating under long-term high-load conditions are prone to initiating micro-cracks on the raceway and rolling elements due to the coupled effects of alternating stress and material fatigue, which may evolve into macroscopic spalling failures under vibration and impact [1]. Industrial statistics indicate that approximately one-third of unplanned downtime in rotating machinery can be attributed to progressive damage in bearing components. The sudden onset of such faults often triggers cascading equipment failures, resulting in single-event economic losses potentially reaching millions. Timely and accurate diagnosis of bearing faults is crucial for extending equipment lifespan, reducing maintenance costs, and ensuring operational safety [2].

Vibration signals are the most commonly used and information-rich medium in bearing condition monitoring. By analyzing and processing vibration signals, various abnormal operating states of bearings can be effectively identified [3,4]. Early bearing fault diagnosis relied on manually extracted features combined with traditional classifiers, such as support vector machines (SVM) [5] random forests (RF) [6] These methods depend heavily on expert knowledge for feature selection and exhibit limited performance when handling complex signals, thereby constraining their applicability in large-scale and complex environments [7]. In recent years, deep learning has emerged as a mainstream approach due to its capabilities for automatic feature extraction and powerful classification performance [8,9]. Complementarily, nonlinear dynamic analysis methods have also gained attention. Techniques such as the time Poincaré plot index (TPPI) and the enhanced hierarchical Poincaré plot index (EHPPI) have demonstrated strong potential for capturing multiscale and nonlinear characteristics in vibration data, with EHPPI further improving diagnostic accuracy by enabling effective multi-sensor signal fusion [10,11]. Li et al. [12] proposed the KANs-CNN-FAN fusion network to extract nonlinear features from bearing signals. Tang et al. [13] developed a convolutional structure based on fusion units that can extract multi-scale features from signals, enabling fault diagnosis with strong generalization ability. Wang et al. [14] proposed a hybrid method combining residual networks and long short-term memory (LSTM) networks, which effectively extracts fault features from bearing signals. Gui et al. [15] introduced the QNN-BiLSTM hybrid model, which integrates the advantages of quadratic neural networks and bidirectional temporal networks to enhance both diagnostic efficiency and model interpretability. Guo et al. [16] designed a compact residual network for cross-domain bearing fault diagnosis by leveraging a teacher network enhanced by DANN-NAM and an IPKD-based quantization-coordinated optimization framework, achieving comparable accuracy with significantly reduced model size.

However, the practical deployment of existing intelligent diagnostic systems still faces significant challenges, primarily due to their strong dependence on the completeness of training data, and their limited generalization capability under domain shifts between different operating conditions. To tackle this challenge, Huang et al. [17] introduced a domain-independent compact boundary learning framework for universal diagnosis domain generation (UDDGN), which addresses arbitrary category shifts and enhances generalization without requiring target domain data. In real-world industrial environments, bearing fault samples are typically scarce, while samples from normal operating conditions are overwhelmingly abundant. This severe class imbalance poses a major challenge for training effective bearing fault diagnosis models [18,19]. Data imbalance tends to bias diagnostic models toward the majority class (i.e., the normal condition), resulting in low recognition rates for minority class faults, reduced generalization performance, and an increased risk of overfitting and misclassification. This issue is particularly pronounced in deep learning models, where neural networks tend to focus on learning the dominant class features while neglecting the subtle variations of minority classes, thereby compromising the sensitivity and accuracy of fault detection [20]. Therefore, effectively mitigating the impact of data imbalance and enhancing the discriminative ability for minority classes remain key bottlenecks in achieving high-performance bearing fault diagnosis.

To address the issue of data imbalance, traditional approaches primarily include resampling techniques (oversampling and undersampling), cost-sensitive learning, and threshold adjustment methods. For example, the Synthetic Minority Oversampling Technique (SMOTE) generates new samples by interpolating within the neighborhood of minority class instances to augment the dataset [21,22]. However, conventional oversampling methods often introduce redundant or noisy samples, leading to insufficient data diversity and limiting model improvement [23]. In contrast, undersampling may result in the loss of critical information, thereby degrading overall model performance. Additionally, cost-sensitive model adjustments often require manually specified parameters, making them difficult to adapt to complex operating conditions [24]. Overall, it is difficult for traditional data augmentation methods to generate samples with similar distribution to the original data.

Data augmentation has become an effective way to address the lack of fault samples in intelligent diagnosis tasks [25]. Among these methods, GAN-based techniques are widely explored for their sample generation capabilities [26]. For instance, Yang et al. [26]proposed a stacked contractive autoencoder combined with an auxiliary classifier GAN (VSC-ACGAN), which improves generation quality and diagnostic accuracy on imbalanced bearing datasets. Liu et al. [27] proposed ACGAN-SG, which integrates spectral normalization and gradient penalty into GANs to generate high-quality samples and reduce class imbalance. Similarly, Qin et al. [28] developed WGAN-GP to improve training stability by penalizing the discriminator’s gradients. Wang et al. [29] enhanced CGAN with spectral normalization to prevent mode collapse and stabilize the training process. To improve generation quality, several studies introduced domain-specific constraints. Ruan et al. [30] incorporated frequency-domain envelope spectrum error into the GAN loss function, guiding the model to generate more realistic fault features. Wang et al. [31] proposed M-D2GAN, which uses a dual-discriminator design and a class-specific generation strategy to handle diverse monitoring data. An additional discriminator was also introduced to address imbalance in normal samples. Other approaches focused on improving interpretability and diversity. Liu et al. [32] designed an interpretable variational autoencoder with sequential attention (AVAE-SQA) to augment imbalanced bearing datasets. Fusion-based models have also shown promise. Zhu et al. [33] presented DWCVAE-DFL, a dynamically weighted conditional variational autoencoder tailored for 3D blade tip clearance signals. Zhu et al. [34] combined Digital Twin technology with C-DCGAN to constrain generation and improve sample fidelity. Yu et al. [35] proposed a method that integrates physical modeling with a CycleGAN variant. By incorporating BiLSTM and multi-head attention, their model can generate fault samples even without real data. Wang et al. [36] used GANs with CNNs to generate balanced datasets for wind turbine SCADA data, enhancing diagnostic accuracy. More recently, Yu et al. [37] introduced ReF-DDPM, a conditional diffusion model that uses noise prediction and label-guided constraints to generate high-quality samples. This method improves both the accuracy and generalization of diagnosis under imbalanced conditions. Similarly, Wang et al. [38] proposed a method combining an improved DDPM with 1D-CNN to enhance small-sample training, effectively improving fault diagnosis performance. Despite these advances, many generative models still focus too heavily on dominant patterns. As a result, they often fail to capture subtle but critical features in real-world data. This leads to limited diversity and reduces the representational capacity of synthetic samples—posing a challenge in replicating the complexity of actual industrial fault scenarios.

**Fig 1:**
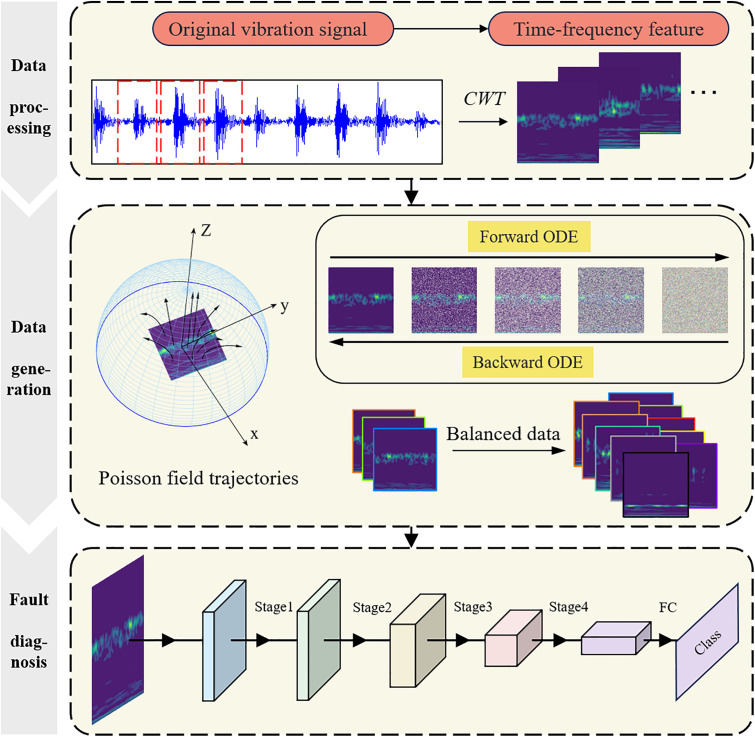
Flowchart of the fault diagnosis based on the proposed PFRNet.

To address imbalanced bearing fault data, we propose PFRNet, a framework based on the Poisson Flow generative model. This approach augments fault data by synthesizing samples that match the statistical patterns of real faults, enabling accurate diagnosis under balanced conditions. Continuous wavelet transform (CWT) is employed to convert the raw vibration signals collected from sensors into time-frequency representations. The Poisson Flow generative model is then used to augment fault samples by generating time-frequency representations that closely match the feature distribution of the original data. Finally, the generated fault samples are combined with real data to construct a balanced dataset, facilitating effective fault diagnosis under data-balanced conditions. The overall methodological framework is illustrated in Fig 1. Experimental results show that, compared with existing approaches, the proposed method achieves higher diagnostic accuracy and effectively handles varying degrees of data imbalance.

The main contributions of this paper can be summarized as follows.

1. A Poisson Flow-based spectrogram generation strategy is proposed to produce high-quality fault samples that preserve essential time-frequency structures and alleviate data imbalance.
2. A compact residual network is developed to efficiently extract both local and global time-frequency features, enhancing diagnostic accuracy with minimal computational cost.
3. An end-to-end framework integrates preprocessing, sample generation, and classification, enabling joint optimization for improved data augmentation and robust fault diagnosis.

The rest of this paper is organized as follows. Section II introduces the basic theoretical background. Section III presents experimental verification of the proposed approach. In Section IV, results are analyzed and compared with state-of-the-art methods. Section V includes the conclusion and research prospect.

## 2. Theoretical Background

### 2.1. Vibration signal collection and preprocessing

**Fig 2:**
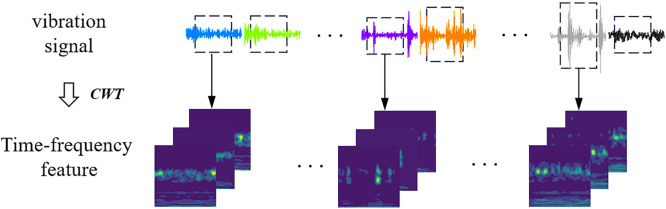
Process for the conversion of time-frequency feature.

In practical operation, rolling bearings are often affected by factors such as load fluctuations, structural nonlinearity, and installation misalignment, leading to non-stationary vibration signals whose frequency components vary over time. This non-stationarity reduces the discriminative capability of traditional time-domain or frequency-domain analysis methods in fault pattern recognition. To more effectively capture the underlying time-varying characteristics of the signals, this study employs Continuous Wavelet Transform (CWT) for time-frequency analysis, enabling dynamic spectral representation of the vibration data. The process of time-frequency feature extraction is illustrated in Fig 2.

CWT exhibits excellent time-frequency localization properties, making it particularly suitable for analyzing impact or transient signals. In this study, the Morlet wavelet, which resembles the shape of impact responses, is selected as the mother wavelet to enhance sensitivity to local features. CWT performs convolution of the signal across different time shifts and scale parameters, enabling a multi-resolution representation of the signal structure. Its mathematical formulation is given as follows:


$$\begin{array}{c} w(\tau ,s)=\frac{\text{1}}{\sqrt{\left|s\right|}}\int x(t)\psi^{*}\left(\frac{t-\tau }{s}\right)dt \end{array}$$ (1)

where x(t) denotes the original signal, τ and s represent the time shift and scale parameters, respectively, and ψ^* (t) is the complex conjugate of the selected wavelet basis function.

The Morlet wavelet used in this study is defined as follows:


$$\begin{array}{c} \psi (t)=\pi^{-\frac{\text{1}}{\text{4}}}\cdot e^{i\omega_{\text{0}}t}\cdot e^{-\frac{t^{\text{2}}}{\text{2}}} \end{array}$$ (2)

where π is the constant pi, i is the imaginary unit, $\omega_{\text{0}}$ is the central angular frequency of the Morlet wavelet, and t represents the time variable.

Through this transformation, the one-dimensional time-series signal is mapped into a two-dimensional time-frequency representation, where the horizontal axis corresponds to time, the vertical axis to frequency, and the pixel intensity indicates energy magnitude. The resulting time-frequency image simultaneously captures local temporal characteristics and frequency distribution features, offering a more discriminative input representation for deep network models.

### 2.2. Data augmentation process

To address the data imbalance issue caused by minority classes in rolling bearing fault diagnosis, this study introduces a Poisson Flow-based generative model to construct high-quality time-frequency feature samples. By incorporating the Poisson generative mechanism into the generation process of bearing time-frequency representations, the model simulates the evolution of sample trajectories in a high-dimensional latent space, thereby enriching the diversity of minority-class samples and mitigating performance degradation due to class imbalance. The core idea is to model samples as points evolving toward the data manifold through learned trajectory dynamics, resulting in the generation of new data instances that match the statistical distribution of real fault features.

The Poisson generative model first maps the original time-frequency sample $x\in R^{N}$ into a high-dimensional augmented space $\tilde{x}=(x,z)\in R^{N+D}$, where $z\in R^{D}$ represents an additional perturbation variable. In this space, samples are constructed via a spherically symmetric perturbation kernel to form the conditional probability distribution $p_{r}(x|y)$, which takes the following form:


$$\begin{array}{c} p_{r}\left(x|y\right)\propto {\left(∥x-y{∥}^{\text{2}}+r^{\text{2}}\right)}^{-\frac{N+D}{\text{2}}} \end{array}$$ (3)

where, $y\in R^{N}$ denotes a sample drawn from the original data distribution p(y), r represents the perturbation radius, and D is the augmented dimensionality parameter introduced to enhance the model’s representational capacity.

To ensure consistency in the shape of the perturbation kernel across different augmented dimensions, the Poisson-based model in this study defines the perturbation radius according to the following proportional scheme:


$$\begin{array}{c} r=\sigma \sqrt{D} \end{array}$$ (4)

where, σ denotes the standard noise intensity.

This configuration ensures scale-aligned properties of the perturbation kernel across different D values—specifically, the intermediate perturbed distribution $p_{r}(x|y)$ remains statistically consistent. Consequently, the geometric structure of the generation path maintains stability and generalizability during dimensional migration. As demonstrated by Xu et al. [39], the model achieves an optimal balance between robustness and generation quality when *D* = 2048, in contrast to extreme diffusion model scenarios. In a 64x64x3 image space, 2048 dimensions can provide sufficient nonlinear modeling capabilities while maintaining the stability of the generation path.

The generation process in the Poisson-based model can be interpreted as the evolution of samples along the streamlines of a normalized electric field, with the direction given by the following integral form:


$$\begin{array}{c} E\left(\tilde{x}\right)=\int \frac{\tilde{x}-y}{∥\tilde{x}-y{∥}^{N+D}}p(y)dy \end{array}$$ (5)

where, $E(\tilde{x})$ represents the electric field direction in the augmented space, and $\tilde{x}=(x,z)$ denotes the current position in this space.

However, due to the difficulty of explicitly solving the electric field function in high-dimensional space, a neural network $f_{\theta }(x)$ is introduced to approximate its direction. The training objective is to make the network output closely align with the sample perturbation direction, and the corresponding loss function is defined as follows:


$$\begin{array}{c} L(\theta )=E_{r,y,x\sim p_{r}\left(x|y\right)}∥f_{\theta }(x)-(x-y)/(r/\sqrt{}D){∥}^{\text{2}} \end{array}$$ (6)

where, $f_{\theta }(x)$ represents the direction vector predicted by the model, and $\frac{x-y}{r/\sqrt{D}}$ denotes the normalized theoretical perturbation direction.

By minimizing the directional loss, the network learns to backtrack the perturbed sample x to its center point y, thereby establishing a reverse flow path from the sample to the target manifold.

After training is completed, the Poisson-based model can initiate from a perturbed point $x_{T}$ far from the data manifold and perform reverse integration along the learned vector field to generate new samples. By integrating the trajectory of an ordinary differential equation (ODE), the process flows back toward the data manifold, enabling sample synthesis. The sampling formula is as follows:


$$\begin{array}{c} x_{t-\text{1}}=x_{t}-\Delta r\cdot \frac{f_{\theta }\left(x_{t}\right)}{∥f_{\theta }\left(x_{t}\right)∥} \end{array}$$ (7)

where, $x_{t}$ denotes the current sampling state, Δr represents the step size that controls the scale of each reverse step, and $∥f_{\theta }(x_{t})∥$ indicates the normalization of the directional vector.

According to the principle of Poisson model, the main parameters for establishing the data generation module are shown in Table 1.

**Table 1:** The key parameters of the Poisson generation module.

Parameter	Setting Value
Input size	(64,64,3)
Original dimension (N)	12288
Augmented dimension (D)	2048
Max perturbation std$\left(\sigma_{\text{max}}\right)$	0.4
Min perturbation std$\left(\sigma_{\text{m}in}\right)$	0.02
Perturbation radius (r)	(0.9, 28.8)
Sampling steps (N)	5
Integration method	Heun’s method (RK2)
Vector field network	Multi-scale U-Net
Optimizer settings	Adam, lr = 2e-4

### 2.3. Fault diagnosis model

In fault feature recognition tasks, although deep networks possess strong feature extraction capabilities, they often encounter performance bottlenecks during training due to gradient vanishing or degradation issues. To address this, this study employs a ResNet architecture, centered on the residual mechanism, as the classifier. This effectively mitigates instability during deep model training and enhances the network’s capacity to model high-dimensional time-frequency features. By introducing identity mapping paths, the residual network allows input features to propagate directly across layers, enabling efficient sharing of critical information at multiple levels, thereby improving the model’s sensitivity to variations in bearing fault patterns.

The fundamental building block of the network is the residual unit, whose core idea is to explicitly learn the difference (residual) between the input and output, rather than directly fitting a complex mapping. For the l-th layer, its structure can be formally expressed as:


$$\begin{array}{c} y_{l}=w_{l}+F\left(w_{l},V_{l}\right) \end{array}$$ (8)

$$\begin{array}{c} w_{l+\text{1}}=\text{ReLU}\left(y_{l}\right) \end{array}$$ (9)

where, $w_{l}$ denotes the input to the l-th layer, and F( ) represents a convolution-normalization-activation composite module with learnable parameters. $V_{l}$ refers to the set of learnable weights associated with the l-th composite module. The intermediate output $y_{l}$ is the residual sum of the input and the transformed features, which is then passed through a ReLU activation to obtain the output $w_{l+\text{1}}$ of the current layer.

The introduction of residual paths allows the model to learn the residual function F via skip connections, preventing the progressive degradation of information in deep networks. To accommodate the time-frequency image inputs generated in this study, a lightweight modification of the original ResNet architecture was implemented. The network structure parameters are shown in Table 2. Detailed descriptions of key training hyperparameters, such as the optimizer, learning rate, and batch size, are provided in Table 3. While maintaining the core residual stacking logic, the number of channels in the initial convolutional kernel was reduced, and excessively deep convolutional layers before the global average pooling were removed to avoid feature loss caused by overfitting or excessive dimensionality reduction. The network output is passed through a Softmax layer to obtain the predicted probabilities for each class, and training is conducted using the standard multi-class cross-entropy loss function:

**Table 2:** Structural parameters of fault diagnosis module.

Layer	Type	Output size
Input	Input Image	64 × 64 × 3
Conv1	Conv + BN + ReLU	64 × 64 × 32
ResBlock1	Residual Block ×2	64 × 64 × 32
MaxPool	Max Pooling	32 × 32 × 32
ResBlock2	Residual Block ×2	32 × 32 × 64
MaxPool	Max Pooling	16 × 16 × 64
ResBlock3	Residual Block ×2	16 × 16 × 128
GlobalAvgPool	Global Average Pooling	1 × 1 × 128
FC	Fully Connected Layer	128
Softmax	Output Probabilities	10

**Table 3:** Hyperparameter Configuration for PFRNet Classification Model Training.

Hyperparameter	Value
Optimizer	Adam
Initial Learning Rate	1e-4
Learning Rate Decay	Cosine Annealing
Batch Size	32
Epochs	100


$$\begin{array}{c} L=-\frac{\text{1}}{N}\sum_{h=\text{1}}^{N}\;\sum_{k=\text{1}}^{C}\;y_{hk}\text{log}\left({\hat{y}}_{hk}\right) \end{array}$$ (10)

where, $y_{hk}\in \{\text{0},\text{1}\}$ denotes the one-hot encoded ground truth label, and ${\hat{y}}_{hk}\in (\text{0},\text{1})$ represents the predicted confidence that sample h belongs to class k. Here, N is the total number of training samples in a batch, and C denotes the number of fault categories. The indices h and k iterate over samples and classes, respectively. By optimizing this loss function in an end-to-end manner, the network can effectively extract critical discriminative features embedded in the time-frequency images, enabling accurate classification and identification of various fault conditions.

## 3. Experiment

The experimental runtime environment uses Python 3.8.10 and Pytorch library. The testbed is equipped with Intel Core i9-14900KF CPU, GeForce RTX4090D GPU 64G RAM.

### 3.1. Data presentation

**Fig 3:**
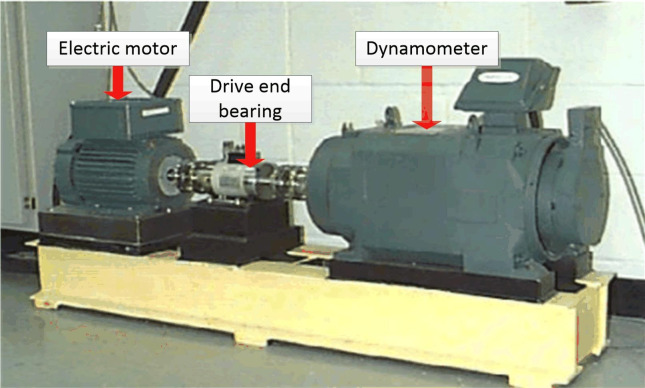
CWRU rolling bearing failure test bed.

The Case Western Reserve University (CWRU) bearing dataset is a widely used benchmark in the field of mechanical fault diagnosis, primarily employed to evaluate the performance of bearing fault detection algorithms. The dataset was collected using the experimental platform shown in Fig 3, and detailed technical specifications of the bearings are provided in Appendix Table 4. The experimental data include vibration signals of the drive-end bearing under normal operating conditions, as well as characteristic vibrations under three typical fault modes: inner race fault, outer race fault, and rolling element fault. For each fault type, three different defect sizes were introduced to simulate varying severities of pitting damage, ranging from mild to severe.

**Table 4:** Bearing parameters.

Parameter	Value
Bearing type	6205−2RS JEM SKF
Pitch circle diameter	39.04 (mm)
Rolling body diameter	7.94 (mm)
Number of scrollers	9
Motor speed	1797/1772/1750/1730 (rpm)
Sampling frequency	12 (kHz)

### 3.2. Data partitioning

To investigate the applicability of bearing fault diagnosis under imbalanced sample distribution scenarios, this study employs data augmentation techniques to mitigate class imbalance in the original dataset. The dataset is divided into training, validation, and test sets in a ratio of 10:1:1.

In the experimental design, we define a dataset imbalance ratio I (see Eq (11)) to quantify varying degrees of sample scarcity, assuming that the imbalance level is consistent across all fault categories. Synthetic samples are used exclusively during the training and validation phases, while the test set consists entirely of original data with an equal number of samples for each fault category. Model training is conducted using the mixed dataset for parameter optimization, and final performance evaluation is carried out on an independent test set. Table 5 provides the specific sample distribution across subsets when the imbalance ratio I is set to 0.9.

**Table 5:** The number of samples when the imbalance degree is 0.9.

Fault type	Train		Valid		Test
	Original	Generated	Original	Generated	Original
B007	50	450	5	45	50
B014	50	450	5	45	50
B021	50	450	5	45	50
IR007	50	450	5	45	50
IR014	50	450	5	45	50
IR021	50	450	5	45	50
OR007	50	450	5	45	50
OR014	50	450	5	45	50
OR021	50	450	5	45	50
Normal	500	/	50	/	50


$$\begin{array}{c} I=\frac{s_{g}}{s_{o}+s_{g}} \end{array}$$ (11)

where, $S_{g}$ represents the number of generated samples, and $S_{o}$ represents the number of original samples.

### 3.3. Experimental results

The proposed model was evaluated on a ten-class dataset alongside CNN, VGGNet, AlexNet, and ViT for classification accuracy. For reproducibility, we briefly describe the main architectural settings of all models, including CNN, AlexNet, VGGNet, ViT, and PFRNet. All models were configured to accept 64 × 64 × 3 time–frequency inputs and produce ten-category outputs. Table 6 summarizes the core components of each model, including major layers and structural parameters. All results are averaged over five independent runs with different random seeds (42, 123, 456, 789, 1000) and reported as mean ± standard deviation. Table 7 reports classification accuracy under varying data imbalance levels.

**Table 6:** Architectural Summary of Compared Models.

Model	Input Size	Conv/Transformer Blocks	Pooling Type	FC Layers	Output Classes	Notes (Modifications/ References)
CNN	64 × 64 × 3	3 × 3 conv ×3 (32–64–128 filters)	MaxPool (2 × 2)	2 (128 → 64 → 10)	10	Custom shallow CNN baseline
AlexNet	64 × 64 × 3	5 conv layers (adapted from ImageNet)	MaxPool	3 (4096 → 4096 → 10)	10	First layer modified for 64 × 64 input [Krizhevsky+12]
VggNet	64 × 64 × 3	VGG-11 style (conv3–conv3–pool ×5)	MaxPool	2 (4096 → 10)	10	Reduced variant of VGG-11 [Simonyan+14]
ViT	64 × 64 × 3	8 Transformer blocks (patch = 4 × 4)		1 (CLS token→10)	10	ViT-Tiny adapted for 64 × 64 input [Dosovitskiy+21]

**Table 7:** Fault diagnosis accuracy of different models (mean±std over 5 runs with different random seeds.

Fault Diagnosis Method	Radio			
	0.8	0.9	0.96	0.98
CNN	90.10% ± 1.20%	88.50% ± 1.50%	86.70% ± 1.80%	84.20% ± 2.10%
AlexNet	97.90% ± 0.80%	96.30% ± 0.95%	95.10% ± 1.10%	94.00% ± 1.30%
VggNet	98.60% ± 0.60%	97.40% ± 0.75%	96.30% ± 0.90%	95.20% ± 1.05%
ViT	98.80% ± 0.50%	98.00% ± 0.65%	96.80% ± 0.80%	95.60% ± 0.95%
PFRNet	99.40% ± 0.32%	98.60% ± 0.45%	97.80% ± 0.55%	96.40% ± 0.70%

Experimental results show that the classification accuracy of all models declines as the class imbalance ratio increases from 0.8 to 0.98, underscoring the persistent challenge of imbalanced data in fault diagnosis. CNN experiences the most significant performance drop, with accuracy decreasing from 90.10% ± 1.20% to 84.20% ± 2.10%, indicating its high sensitivity to class distribution skew. In contrast, AlexNet and VGGNet exhibit greater resilience. At an imbalance ratio of 0.98, AlexNet achieves 94.00% ± 1.30%, while VGGNet reaches 95.20% ± 1.05%, reflecting the advantages of deeper convolutional architectures in handling data imbalance and random variation. ViT, a Transformer-based classifier, consistently outperforms VGGNet across all scenarios, with accuracy decreasing modestly from 98.80% ± 0.50% at 0.8 to 95.60% ± 0.95% at 0.98. Its lower standard deviations further highlight strong generalization capability.

The proposed PFRNet attains the highest accuracy across all imbalance levels, achieving 99.40% ± 0.32% at a ratio of 0.8 and 96.40% ± 0.70% at 0.98. Notably, it yields the smallest standard deviations across all settings, indicating strong robustness to both initialization and data variation. These results suggest that combining Poisson Flow-based augmentation with a lightweight residual network allows PFRNet to address class imbalance more effectively than conventional CNNs and Transformer-based models.

**Fig 4:**
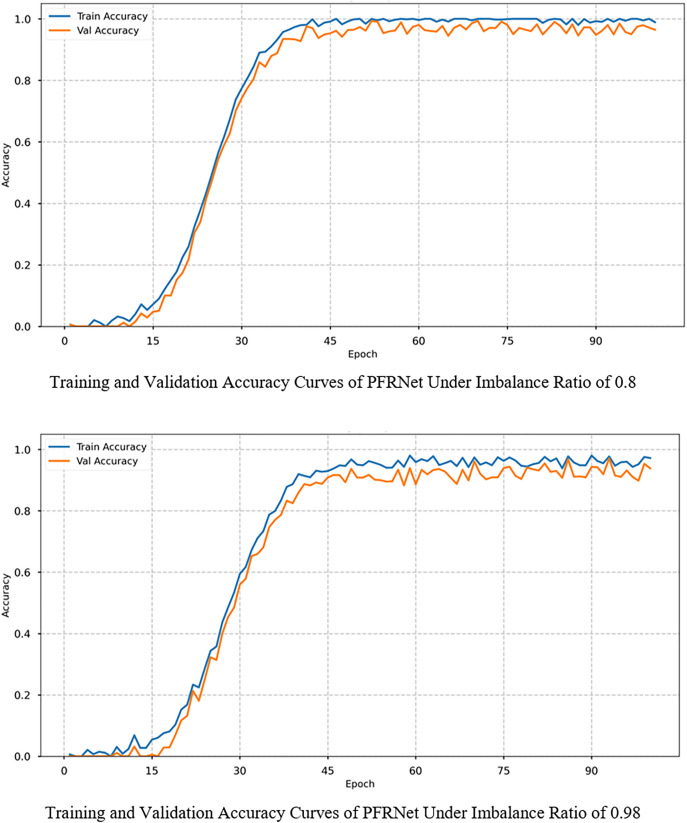
Training and validation accuracy curves of PFRNet under different imbalance ratios.

To further validate the training stability of PFRNet, Fig 4 displays training and validation accuracy curves under imbalance ratios of 0.8 and 0.98. In both cases, the model converges rapidly within 30 epochs, with training and validation curves increasing synchronously and maintaining narrow gaps. This demonstrates that the selected hyperparameters (Table 3) enable stable learning while avoiding overfitting under different imbalance conditions.

## 4. Analysis

### 4.1. Confusion matrix and class-wise metric analysis

**Fig 5:**
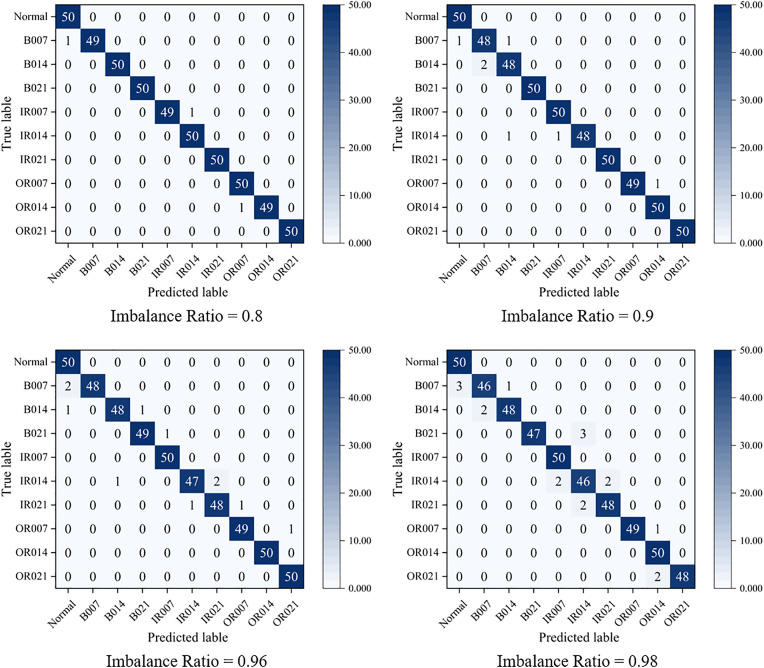
Confusion matrix of PFRNet model test set under different data balance ratios.

Fig 5 illustrate the confusion matrices of the proposed PFRNet model on the test set under different data balance ratios (0.8, 0.9, 0.96, and 0.98). The results indicate that PFRNet demonstrates strong fault identification capabilities across all balance ratios. Particularly under relatively balanced conditions (e.g., ratios of 0.8 and 0.9), the model achieves high accuracy for all fault categories, with diagonal elements in the confusion matrix approaching ideal values, suggesting excellent feature extraction and discrimination performance. Even under highly imbalanced conditions (balance ratio of 0.98), PFRNet maintains stable recognition for most classes, with only slight misclassifications in minority categories, and still outperforms conventional deep networks overall. These findings highlight the robustness and generalization capability of PFRNet; however, extreme imbalance can still hinder the model’s ability to recognize minority classes, underscoring the importance of data balancing in improving diagnostic performance.

To further evaluate PFRNet’s class-wise performance under imbalanced conditions, Table 8 reports precision, recall, and F1-scores across four imbalance ratios (0.8–0.98). Most fault categories (e.g., B021, IR021, OR014) achieve near-perfect metrics (>98%) across all scenarios, indicating robust feature discrimination. For imbalance-sensitive minority classes (e.g., B007, IR014), slight performance declines are observed under extreme imbalance (ratio = 0.98); however, their F1-scores remain above 93%, confirming effective mitigation of under-recognition. The majority class (Normal) also maintains high stability (F1-score ≥ 97.07%), indicating that PFRNet avoids bias toward dominant categories. These results demonstrate PFRNet’s superior class-specific discriminative capability under imbalanced conditions.

**Table 8:** Class-wise precision, recall, and F1-score of PFRNet under different imbalance ratios (%).

	Preci-sion(%)				Recall(%)				F1-Score(%)			
	0.8	0.9	0.96	0.98	0.8	0.9	0.96	0.98	0.8	0.9	0.96	0.98
Normal	98.04	98.04	94.34	94.34	100	100	100	100	99.01	99.01	97.07	97.07
B007	100	96	100	95.83	98	96	96	92	98.99	96	97.96	93.8
B014	100	97.96	97.96	100	100	96	97.96	96	100	96.95	97.96	97.96
B021	100	100	100	100	100	100	98	94	100	100	98.99	96
IR007	100	100	100	96.15	98	100	100	100	98.99	100	100	98.05
IR014	100	100	97.92	95.83	100	96	97.92	92	100	97.96	97.92	93.8
IR021	100	100	100	100	100	100	97.96	96	100	100	98.97	97.96
OR007	100	100	100	100	100	98	98	98	100	98.99	98.99	99
OR014	98	100	100	100	100	100	100	100	98.99	100	100	100
OR021	100	100	100	96	100	100	100	96	100	100	100	96

**Fig 6:**
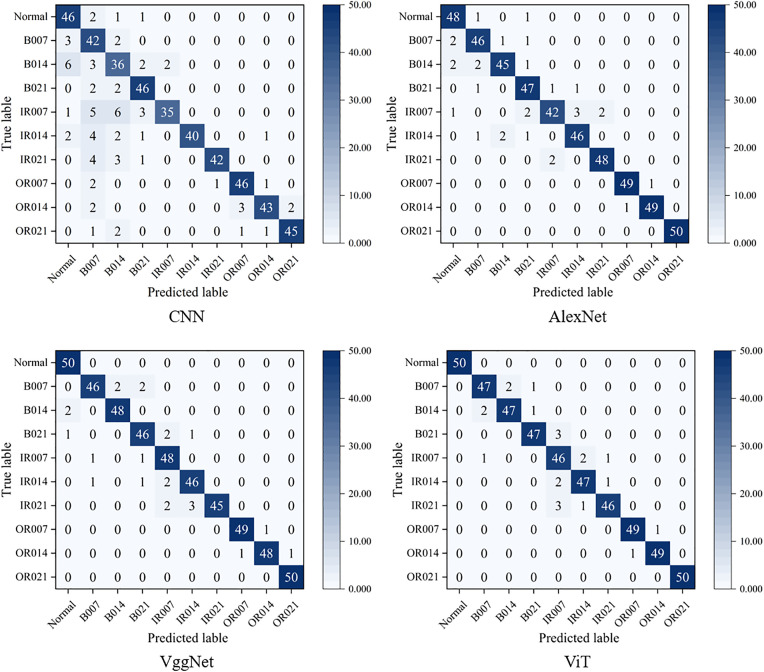
Confusion matrix for the comparison model test set at imbalance 0.98.

Fig 6 display the confusion matrices for CNN, AlexNet, VggNet, and ViT at an imbalance ratio of 0.98. As data imbalance increases, all these models exhibit degraded performance. The traditional CNN shows high misclassification rates across categories, with severe confusion between IR007 and IR014, indicating poor recognition of minority classes. Although AlexNet and VggNet achieve higher accuracy than CNN for some faults, they still misclassify minority categories such as OR014 and B014. ViT despite some diagonal concentration in its confusion matrix, misclassifies minority cases and lacks consistent stability in predicting them. Notably, PFRNet outperforms all other models across most fault categories, exhibiting a more concentrated diagonal in the confusion matrix, which indicates more accurate and stable predictions. Even under extreme data imbalance, PFRNet accurately identifies typical faults such as IR007, IR021, and OR021, demonstrating strong feature extraction capabilities and adaptability to minority classes. These findings further confirm the robustness and practical value of PFRNet in real-world imbalanced data scenarios.

### 4.2. Comparison of the effects of different data generation models

Table 9 presents the ablation results that quantify the individual contributions of data augmentation and classifier modules to diagnostic performance under imbalanced conditions. These results clearly demonstrate a positive correlation between diagnostic accuracy and the advancement of data augmentation strategies, underscoring the essential role of high-quality sample generation in mitigating class imbalance.

**Table 9:** Comparing experimental results.

Data Enhancement	Classifier	Radio			
		0.8	0.9	0.96	0.98
None	CNN	80.50%	78.20%	75.10%	72.60%
	AlexNet	89.30%	87.80%	85.40%	80.10%
	VggNet	90.80%	89.50%	86.70%	82.30%
	ResNet	91.60%	90.20%	88.90%	83.70%
GAN	CNN	84.20%	82.40%	80.80%	78.50%
	AlexNet	93.50%	91.20%	89.60%	86.90%
	VggNet	94.10%	92.70%	90.30%	88.10%
	ResNet	94.90%	93.80%	91.50%	89.40%
DCGAN	CNN	86.70%	84.10%	82.90%	81.30%
	Alexnet	95.10%	93.60%	91.80%	89.70%
	VggNet	95.80%	94.20%	92.50%	90.60%
	ResNet	96.40%	95.30%	93.10%	91.80%
BAGAN	CNN	87.50%	85.00%	83.20%	80.80%
	AlexNet	95.90%	94.10%	92.50%	90.20%
	VggNet	96.30%	94.80%	93.40%	91.50%
	ResNet	97.00%	95.70%	94.00%	92.30%
DDPM	CNN	88.20%	85.90%	84.30%	79.80%
	AlexNet	96.30%	94.80%	93.20%	91.50%
	VggNet	97.10%	95.60%	94.10%	92.70%
	ResNet	97.80%	96.40%	95.20%	93.90%
DDIM	CNN	89.00%	86.50%	85.00%	85.00%
	AlexNet	96.80%	95.20%	93.80%	93.80%
	VggNet	97.50%	96.00%	94.70%	94.70%
	ResNet	98.20%	96.80%	96.80%	95.80%
TransGAN	CNN	88.50%	87.00%	85.50%	82.00%
	AlexNet	97.00%	95.50%	94.00%	92.50%
	VggNet	97.60%	96.20%	95.20%	93.50%
	ResNet	98.30%	97.00%	96.00%	94.80%
PFRNet	\	99.40%	98.60%	97.80%	96.40%

As the ablation baseline, the “No Augmentation” group (without any data augmentation) reveals that all classifiers suffer performance degradation when trained on raw imbalanced data. For instance, CNN achieves only 72.60% accuracy at an imbalance ratio of 0.98, while ResNet, despite its stronger feature extraction ability, achieves only 83.70% under the same condition. This significant performance gap between the baseline and augmented groups strongly confirms that data augmentation is indispensable for addressing class imbalance.

Among traditional generative approaches, GAN and its variants (DCGAN, BAGAN) enhance diagnostic accuracy over the baseline, but are outperformed by diffusion-based models. DDPM, a foundational diffusion model, achieves 93.90% accuracy with ResNet at an imbalance ratio of 0.98. Its optimized variant, DDIM, further improves this to 95.80% due to more efficient sampling. TransGAN, which integrates Transformer architecture with GAN, outperforms conventional GANs—for example, achieving 94.80% accuracy with ResNet at I = 0.98—by leveraging long-range feature modeling, but still falls short compared to diffusion-based methods.

The proposed PFRNet, as the core ablation target, outperforms all alternative augmentation modules across all imbalance levels, achieving 99.40% accuracy at I = 0.8 and 96.40% at I = 0.98. This superior performance stems from two key components validated by ablation analysis: (1) the Poisson Flow-based generative mechanism, which synthesizes minority-class samples that are statistically consistent with real data; and (2) the lightweight residual network, which efficiently extracts discriminative features from time-frequency representations. These results confirm that PFRNet’s integrated framework provides synergistic benefits beyond those of individual components, demonstrating its effectiveness in addressing imbalanced fault diagnosis.

### 4.3. Visual analysis of T-SNE features

**Fig 7:**
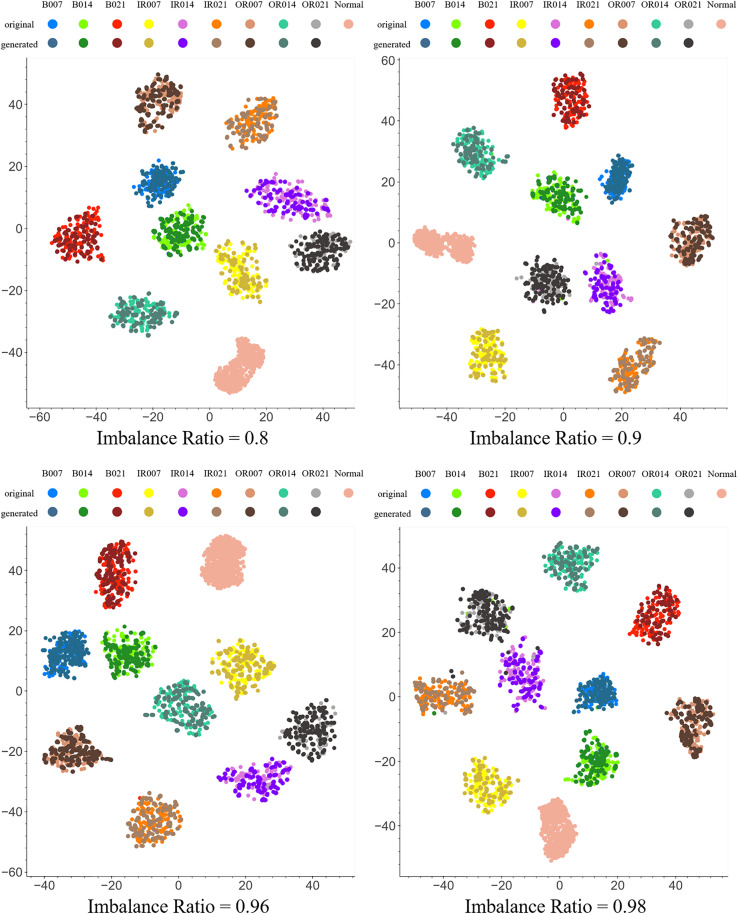
T-SNE feature visualization of the PFRNet model generated data and the original data with different balance degrees.

To provide a more intuitive assessment of the PFRNet model’s performance in data generation tasks—particularly its capability for class discrimination in high-dimensional feature space and its impact on feature learning for fault diagnosis—this study employs the t-Distributed Stochastic Neighbor Embedding (t-SNE) algorithm to visualize the distribution of both generated and real samples in the feature space. As a nonlinear dimensionality reduction technique, t-SNE effectively preserves local structural relationships among high-dimensional features and is well-suited for cluster visualization in complex feature spaces.As illustrated in Fig 7, the generated samples exhibit strong cohesion with real samples of the same class in the low-dimensional space, and distinct, well-separated clusters are formed among different fault categories. This observation indicates that the samples generated by PFRNet are highly consistent with the real data in terms of feature representation, accurately simulating the original distribution patterns of various fault types and thereby enhancing the generalization capability of the diagnostic model.Even under extremely imbalanced data conditions (imbalance ratio = 0.98), where slight overlaps among feature points of certain minority classes are observed, the overall clustering structure remains well separated. These findings further confirm the robustness and discriminative capacity of the proposed model in handling severe class imbalance. The generated samples not only effectively augment minority class data but also maintain strong inter-class separability in the feature space.In summary, the t-SNE visualization results clearly demonstrate that the samples generated by PFRNet offer significant advantages in preserving feature distribution consistency and enhancing fault class discriminability.

### 4.4. Generate data quality analysis

**Fig 8:**
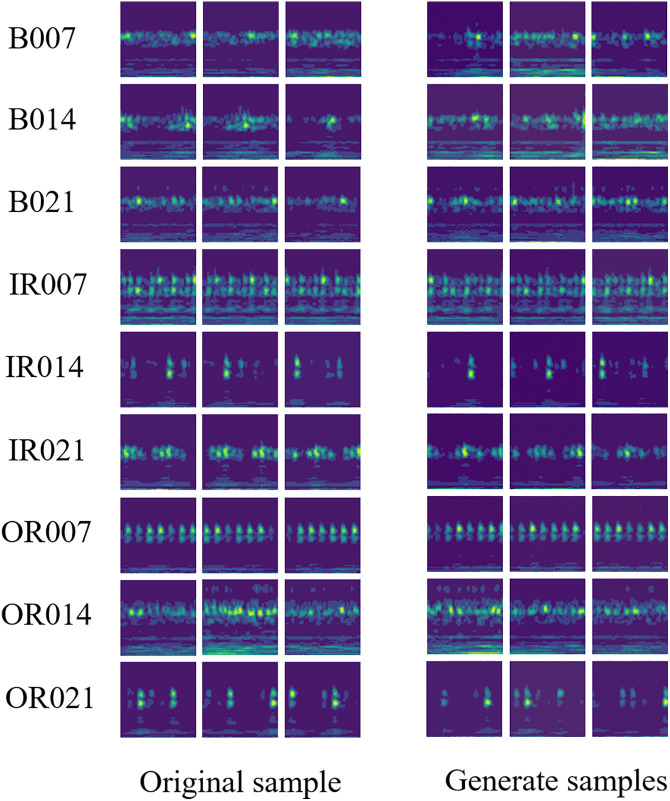
Comparison of Time – Frequency Representations between Original Samples and Generated Samples for Different Fault Types.

As shown in Fig 8, we compare the time–frequency representations of original samples with those generated via Poisson Flow-based data augmentation across various fault types. For instance, in cases such as B007 and B014, the generated samples exhibit time–frequency feature distributions that closely align with the originals. In terms of energy concentration, temporal distribution of impact signals, and frequency band patterns, the augmented data effectively replicates the key structural characteristics of the original signals. This provides intuitive visual evidence supporting the effectiveness of the Poisson Flow-based augmentation method. By preserving fault-relevant time–frequency features, the generated samples demonstrate strong potential as reliable and informative inputs for imbalanced fault diagnosis tasks.

To evaluate the generative model’s ability to simulate real fault data, three widely used metrics are adopted: Fréchet Inception Distance (FID), Inception Score (IS), and Maximum Mean Discrepancy (MMD), providing a comprehensive assessment of similarity between real and generated samples.

**Fig 9:**
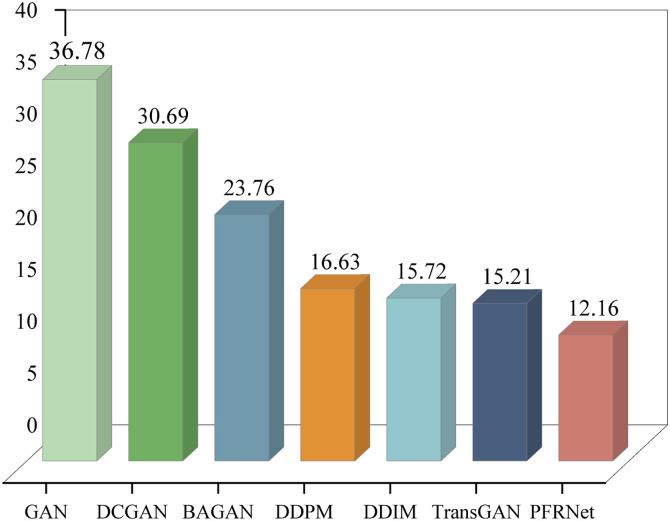
Comparison of data FID values generated by different data generation models.

FID measures the distance between Gaussian distributions fitted to Inception-v3 features of real and generated data:


$$\begin{array}{c} \text{FID}=∥\mu_{r}-\mu_{g}{∥}^{\text{2}}+\text{Tr}(\Sigma_{r}+\Sigma_{g}-\text{2}\left(\Sigma_{r}\Sigma_{g}{)}^{\frac{\text{1}}{\text{2}}}\right) \end{array}$$ (12)

where μ and Σ denote feature means and covariances from the pool3 layer. Fig 9 shows that PFRNet achieves lower FID, indicating better alignment with real data.

**Fig 10:**
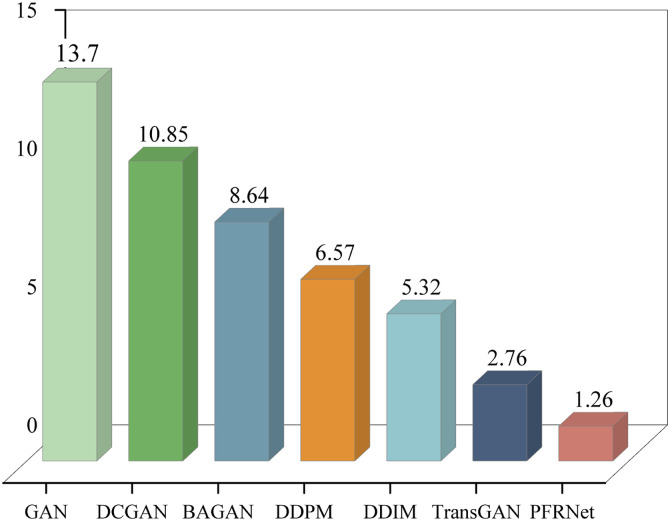
Comparison of MMD values of data generated by different data generation models.

MMD quantifies distributional differences using kernel embeddings:


$$\begin{array}{c} {\text{MMD}}^{\text{2}}=E\left[k\left(x,x^{\prime }\right)\right]+E\left[k\left(y,y^{\prime }\right)\right]-\text{2}E\left[k(x,y)\right] \end{array}$$ (13)

where x and y represent real and generated samples, and k(,) is the Gaussian kernel. The bandwidth parameter was selected using the median heuristic. As shown in Fig 10, PFRNet attains the lowest MMD in most categories, suggesting strong distributional consistency.

**Fig 11:**
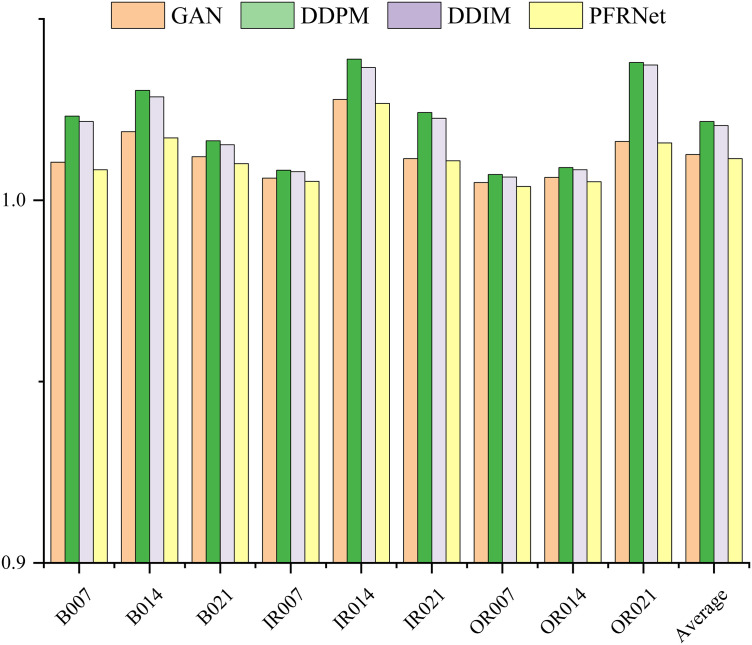
Comparison of data IS values generated by different data generation models.

Inception Score (IS) evaluates both the clarity and diversity of generated samples based on the conditional label distribution predicted by Inception-v3. It is computed as:


$$\begin{array}{c} \text{IS}=\text{exp}(E_{x}\left[\text{KL}\left(p\left(y|x\right)|\left|p(y)\right)\right]\right) \end{array}$$ (14)

where p(y|x) is the predicted label distribution for a generated sample x, and p(y) is the marginal class distribution. IS was calculated using 10 splits, following the standard protocol. The results in Fig 11 show that the proposed model consistently achieves the highest IS across all fault categories, outperforming the competing approaches.

In summary, the evaluation results based on the three metrics consistently demonstrate that the proposed PFRNet model outperforms baseline models in terms of sample realism, distributional similarity, and diversity, thereby confirming its effectiveness and robustness in fault sample generation tasks.

## 5. Conclusions

This study proposes a fault diagnosis method that integrates continuous wavelet transform with a Poisson-based sample generation mechanism to address the issue of imbalanced data distribution in rolling bearing fault diagnosis. The method extracts time-frequency features from raw signals using continuous wavelet transform and generates samples for underrepresented fault categories based on a Poisson distribution model, thereby improving both data balance and sample diversity. Validation on the CWRU bearing dataset demonstrates that the proposed approach can generate high-quality fault samples, significantly enhancing classification accuracy, stability, and robustness under various imbalance scenarios. These results highlight the potential of the method for practical application and industrial deployment.

It should be noted that the present validation is conducted on the CWRU dataset under controlled laboratory conditions. In real industrial environments, domain shifts and variations in operating conditions, such as fluctuating loads, variable speeds, and environmental noise, may influence the generalizability of the proposed framework. Future research will therefore focus on evaluating its performance on diverse industrial datasets and exploring domain adaptation strategies to enhance robustness in practical deployments.
